# Supplementary figures and images for: Transcriptome and Metabolome Profiling to Explore the Causes of Purple Leaves Formation in Non-Heading Chinese Cabbage (Brassica rapa L. ssp. chinensis Makino var. mutliceps Hort.)
Source: Foods. 2022 Jun 17;11(12):1787. doi: 10.3390/foods11121787 (PMC9222747; doi:10.3390/foods11121787)

### Figure S1

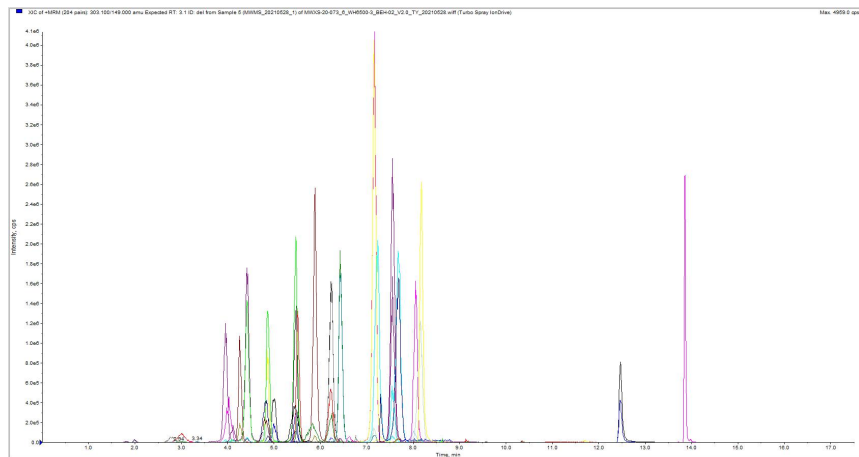

Supplement: Supplementary file 1 [file foods-11-01787-s001.zip › foods-1720527-supplementary/Figure S1.pdf]
